# A Case Series on the Combined Use of Zinc L‐Carnosine and Nd:YAG Photobiomodulation for Radiation‐Induced Oral Mucositis: Pain Reduction and Lesion Improvement

**DOI:** 10.1155/crid/7025093

**Published:** 2026-04-03

**Authors:** Pierpaolo De Francesco, Paolo Vescovi, Claudia Grondelli, Carmen Mortellaro, Ilaria Giovannacci

**Affiliations:** ^1^ Department of Medicine and Surgery, University of Parma, Parma, Italy, unipr.it; ^2^ Department of Radiation Oncology and Radiosurgery, University Hospital of Parma, Parma, Italy, parmahospital.org; ^3^ UniCamillus, Saint Camillus International University of Health Sciences, Rome, Italy, unicamillus.org

**Keywords:** case report, Nd:YAG laser photobiomodulation, oral pathology, radiation-induced oral mucositis, zinc L-carnosine

## Abstract

Radiation‐induced oral mucositis (RIOM) is a severe complication in patients with head and neck cancer (HNC) undergoing radiotherapy (RT). Despite several proposed strategies for prevention and treatment, the results remain inconsistent and unpredictable. A combined approach with zinc L‐carnosine (ZnC) mouthwash and Nd:YAG laser photobiomodulation (PBM) was evaluated in three patients affected by RIOM, treated at the Unit of Oral Medicine, Pathology and Laser‐assisted Surgery of the University of Parma, Italy. One patient had oropharyngeal cancer, one had tongue cancer, and one had soft palate cancer. Two patients presented with World Health Organization (WHO) grade III mucositis, and one with WHO grade II mucositis. The percentage reduction in oral lesions was calculated using Fiji software. All patients reported clinical improvement and a reduction in algic symptoms. A significant decrease in pain was observed after only 2 days, while complete mucosal healing was achieved within 3 weeks. These preliminary results suggest that the combined use of ZnC and Nd:YAG laser PBM may be a promising therapeutic option for patients affected by RIOM, warranting further investigation in larger clinical studies.

## 1. Introduction

Patients with head and neck cancer (HNC) currently represent one of the most complex clinical challenges globally, characterized by high incidence and mortality rates and often requiring a multidisciplinary approach with an estimated overall 5‐year survival rate of around 40% [1]. The therapeutic approach is multimodal and depends on numerous clinical factors, but radiotherapy (RT) plays a crucial role in the management of patients affected by HNC. However, the effects of RT are often not limited to the tumor but can also have damaging effects on surrounding tissues. Consequently, patients with HNC undergoing RT often develop radiation‐induced oral mucositis (RIOM), with a prevalence that increases when chemotherapy is administered concurrently [2]. RIOM is characterized by inflammation of the oral cavity, which can lead to ulcerations and erosions of the oral mucosa, causing intense pain, difficulty eating, and a negative impact on quality of life. In severe cases, it may be necessary to interrupt or modify cancer treatment [3, 4]. RIOM management remains a difficult challenge. Currently, there is no universally recognized therapeutic strategy, and management is largely based on oral care support measures [5]. The aim of this paper is to describe a new promising approach that combines the advantages of laser photobiomodulation (PBM) and a mouthwash based on zinc L‐carnosine (ZnC). To our knowledge, this is the first case series describing the combined use of ZnC mouthwash and Nd:YAG PBM for the management of RIOM.

### 1.1. ZnC

ZnC is a chelated complex of zinc and L‐carnosine that exerts both anti‐inflammatory and antioxidant effects, thereby supporting mucosal protection and repair [6, 7]. Experimental evidence suggests that ZnC has a high potential for modulating the inflammatory response, limiting oxidative stress, and promoting the synthesis of growth factors involved in tissue repair [6].

Zinc is an essential trace mineral, and its presence is fundamental for enzymes such as DNA and RNA polymerase, processes that are essential during tissue regeneration [8]. Numerous studies have shown that the antioxidant and anti‐inflammatory action of ZnC depends mainly on its zinc content [9]. L‐Carnosine is a dipeptide consisting of two amino acids, L‐histidine and β‐alanine, and is mainly present in skeletal muscles. It has antioxidant properties, which are useful in wound healing and immune function. Furthermore, there is in vitro evidence of the anti‐inflammatory properties of carnosine. In particular, the expression of IL‐8 induced by TNF‐a in intestinal epithelial cells was suppressed in its presence [10]. Together, these mechanisms contribute to the restoration of epithelial integrity by promoting cell migration and proliferation, which are key events in tissue healing [11]. Furthermore, several studies in the literature have highlighted a beneficial effect of ZnC, associated with a reduction in the incidence and severity of RIOM in patients undergoing RT for HNC, when used as a preventive treatment [12, 13].

### 1.2. Laser PBM

Low‐level laser therapy (LLLT), also known as laser PBM, has been widely demonstrated to accelerate oral mucosal wound healing by promoting angiogenesis and the release of growth factors [14–16]. Experimental and clinical studies suggest that PBM may facilitate tissue repair through modulation of cellular metabolism and inflammatory pathways, promoting better tissue healing and improved symptom recovery [14]. Scientific evidence shows a beneficial effect of PBM in the management of patients with RIOM, with a reduction in the incidence and severity of the disease in both pediatric and adult cancer patients. PBM is a noninvasive intervention that can offer benefits in terms of both quality of life and treatment adherence [17, 18].

The present case series evaluated the efficacy in lesions improvement and in pain reduction of a combined approach involving the use of a ZnC mouthwash and Nd:YAG laser PBM in three patients affected with HNC that developed RIOM.

## 2. Case Presentation

This study was conducted in compliance with the ethical guidelines of the Academic Hospital of Parma. The treatment was carried out in accordance with the principles of the Declaration of Helsinki. Ethical approval by area Vasta Emilia Nord Ethics Committee (Parma) granted authorization for the study (Protocol Number 40780; Code N umber 500/2025/PARERE/AOUPR; September 29, 2025).

**Figure 1:**
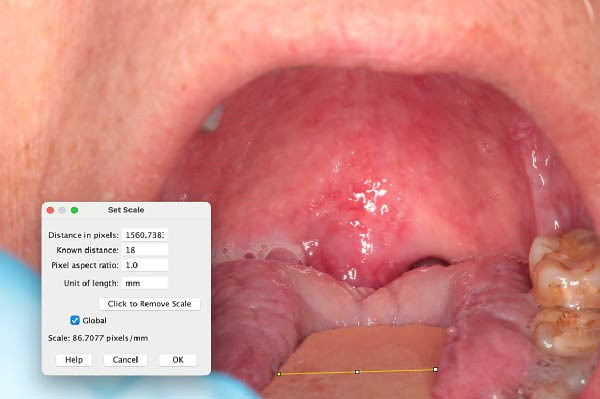
Image calibration performed using a known reference distance (18 mm, tongue depressor width) to allow standardized and reproducible calculation of the inflamed mucosal surface area.

At the Center for Oral Medicine and Laser Surgery at the University of Parma, Italy, three patients affected with HNC treated with RT developed RIOM. The OM stage was assessed on the basis of World Health Organization (WHO) classification (Table 1). Pain evaluation was performed using the visual analog scale (VAS) and numerical rating scale (NRS) on day 0, 2, 7, and 14 after starting OM treatment. The extent of the oral lesions was quantified using Fiji software (ImageJ, NIH, USA). The extent of pathological area was measured in mm^2^, by using a known reference measurement (the width of the tongue depressor—18 mm) for calibration (Figure 1). This calibration allowed for accurate calculation of the inflamed area before and after treatment (Figure 2a,b).

Figure 2Case 1: quantitative assessment of ulcerative area. (a) Baseline (T0): extensive ulcerative lesion involving the soft and hard palate mucosa, WHO grade III mucositis (measured area: 934 mm^2^). (b) Follow‐up at T14: marked reduction in ulcerative surface (94 mm^2^) after combined treatment with ZnC mouthwash and Nd:YAG PBM (WHO grade I).

**Figure:**
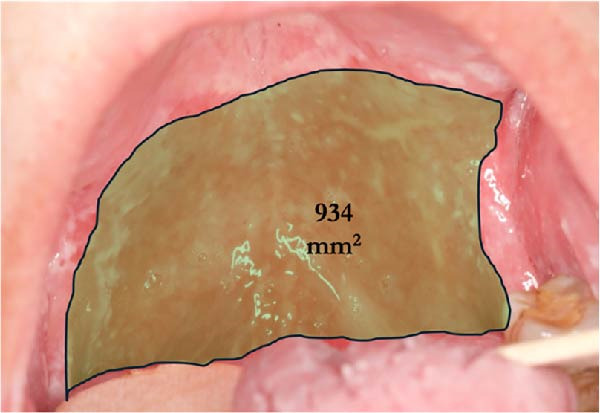
(a)

**Figure:**
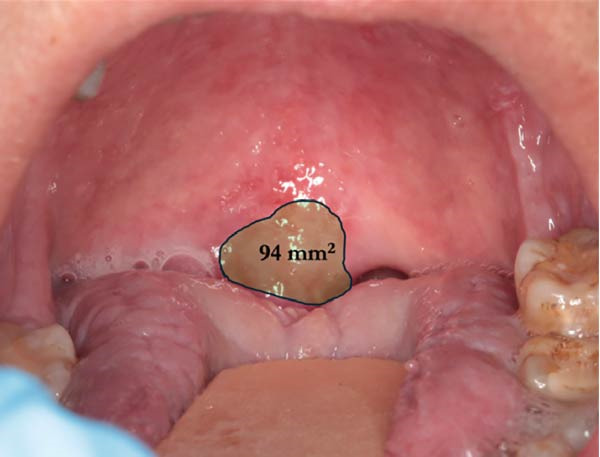
(b)

**Table 1:** WHO classification.

Grade	Clinical aspects
0	No clinical signs
1	Erythema and pain; no ulceration
2	Erythema, ulcers, solid diet tolerated
3	Oral ulcers, only liquid diet
4	Inability to tolerate a solid or liquid diet

### 2.1. RT Protocol

All patients were placed in a supine position with a neutral neck immobilized with a custom‐fitted mask; the planning CT was acquired from vertex to tracheal bifurcation with 3 mm slices and intravenous contrast (CT unit Philips Brilliance Big Bore).

Planning volumes were delineated according to the ICRU definition, and treatment planning performed with Varian Eclipse, VMAT_IG technique, 6 MV photon beams from Varian True beam Linac or Halcyon. Daily set up verification was used with cone beam CT.

Patients with HNCs, treated with adjuvant RT received a total dose of 60–66 Gy (2 Gy/fraction) to surgical bed or flap, high‐risk regions, and lymph node neck levels, and 54 Gy (2 Gy/fraction) for low‐risk neck; concomitant chemotherapy was used in case of extranodal extension or R1.

RT as primary treatment for HNCs (nasopharynx, oropharynx, hypopharynxor, larynx, salivary gland tumors, and occult primary), associated with chemotherapy according to stage indication, is performed with a total dose of 70 Gy (2 Gy/fraction) or 69.96 Gy (2.12 Gy/fr) to primary tumor and involved nodes; 60 Gy (2 Gy/fraction) or 59.4 (1.8 Gy/fraction) for high‐risk regions and 54 Gy for low‐risk regions (1.64–2 Gy/fraction).

Clinical examination was planned for all patients once a week during treatment or in any case of treatment‐related symptom occurrence, monitoring for mucositis mycosis dysphagia, pain, and weight loss.

### 2.2. Case Report

#### 2.2.1. Case 1

A 65‐year‐old woman, smoker, hypertensive, affected with squamous cell carcinoma (pT2N2) of the tonsil, developed a WHO grade III mucositis during head and neck adjuvant RT and chemotherapy. The patient was receiving nebivolol and acetylsalicylic acid for the management of arterial hypertension. An intraoral examination (Figure 2a) revealed an erythematous and ulcerated area on the hard and soft palate. Diffuse plaque accumulation and generalized calculus deposits were observed at baseline. The patient reported intense pain, difficulty eating solid foods, and dysgeusia during meals. The patient reported the onset of oral symptoms ~10 days prior to clinical diagnosis of RIOM. The combined treatment with ZnC and Nd:YAG PBM was initiated immediately after the clinical diagnosis.

#### 2.2.2. Case 2

An 82‐year‐old woman, nonsmoker, with a medical history of thyroid goiter, hypertension, and acute diverticulitis, affected by left maxillary squamous cell carcinoma (pT4aN0M0), developed a WHO grade III mucositis during adjuvant RT. Her past surgical history included right femoral neck fracture treated with orthopedic surgery in 2016, bilateral knee arthroplasty in 2021, and right saphenectomy in 2021.

An intraoral and extraoral examination (Figure 3) revealed the presence of erosive and ulcerative areas involving the left labial commissure, the left lateral border of the tongue, and the left hard and soft palate. The patient reported no severe pain but an inability to ingest solid foods. Oral hygiene status at baseline was considered good, with no significant plaque accumulation or calculus deposits. Oral symptoms had been present for ~7 days before the patient was evaluated in our unit, where RIOM was clinically confirmed. The combined therapeutic protocol was initiated immediately after clinical diagnosis.

Figure 3Case 2: baseline (T0): (a and b) erosive and ulcerative areas involving the left labial commissure; (c) left hard and soft palate; (d) lateral border of the tongue. WHO grade III mucositis.

**Figure:**
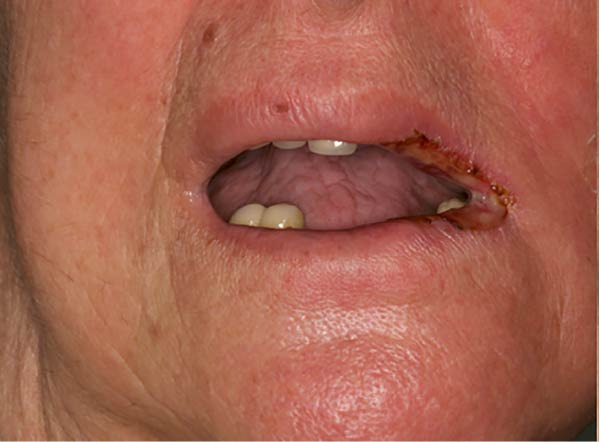
(a)

**Figure:**
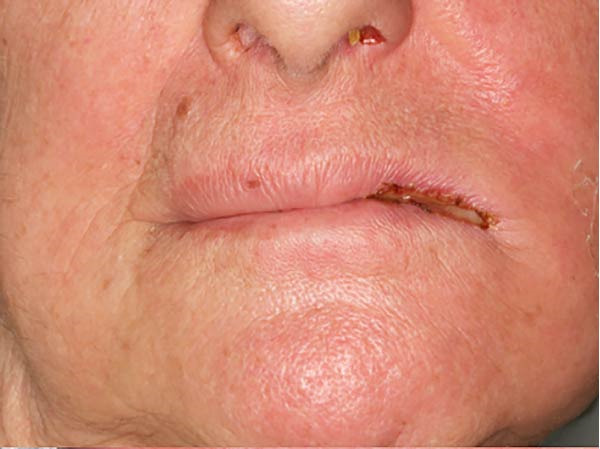
(b)

**Figure:**
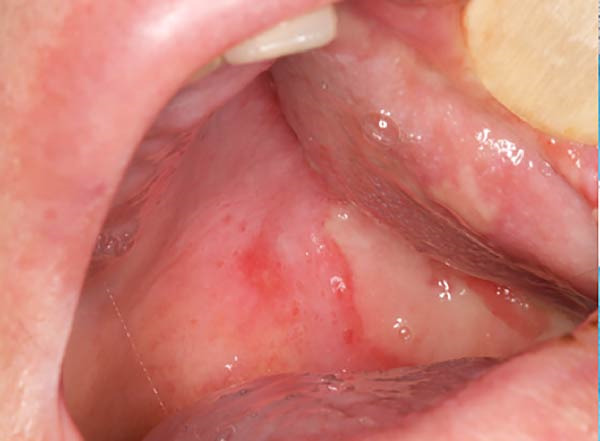
(c)

**Figure:**
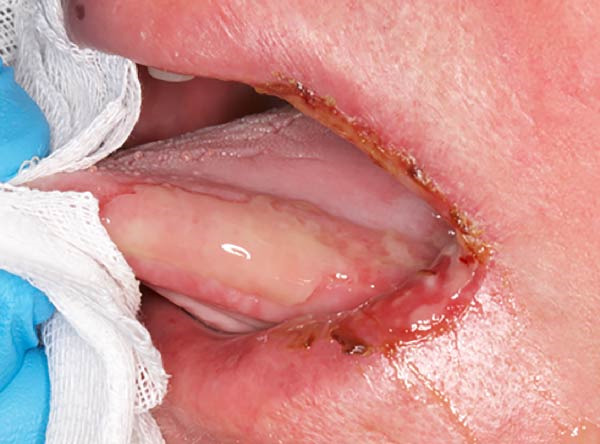
(d)

#### 2.2.3. Case 3

A 51‐year‐old man, smoker (20 cigarettes per day), affected by keratinizing squamous cell carcinoma (pT3N0M0) of the left floor of the mouth, developed a WHO grade II mucositis during head and neck adjuvant RT. His past medical history included inguinal hernioplasty in 2012 and cataract surgery in 2024.

An intraoral examination revealed the presence of erythematous areas on the right and left buccal mucosa (Figure 4). The patient reported significant pain. Oral hygiene at baseline was poor, with diffuse periodontal disease and evident plaque accumulation. As soon as oral symptoms appeared during RT, the patient was referred to our unit. At the time of the visit, a clinical diagnosis of RIOM was made and the combined treatment protocol was initiated.

Figure 4Case 3: baseline (T0): intraoral examination shows erythematous areas on the right (a) and left (b) buccal mucosa. WHO grade II mucositis.

**Figure:**
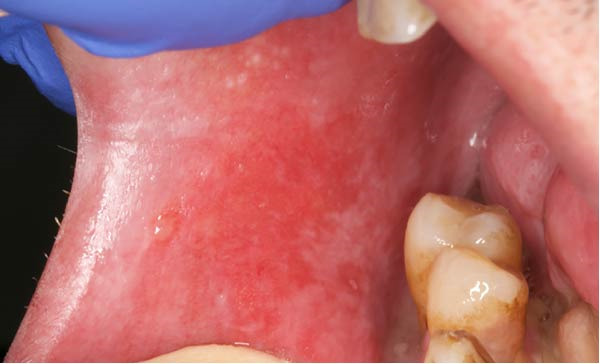
(a)

**Figure:**
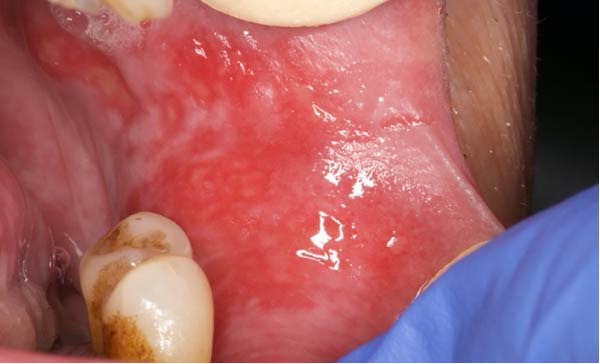
(b)

### 2.3. Treatment Protocol

After OM diagnosis, patients were treated with a combined therapeutic approach, taking advantage of Hepilor mouthwash (Azienda Farmaceutica Italiana s.r.l., S. Egidio alla Vibrata, TE, Italy) and advantages of laser PBM. PBM applications were performed using Nd:YAG laser (1064 nm, FidelisPlus, Fotona, Ljubljana, Slovenia, 1.25 W, 15 Hz).

Laser treatment started on the day of diagnoses and continued once a week for 4 weeks. Each PBM session was performed with a 320 µm fiber, delivering laser light in nonfocused mode at a distance of 2 mm from the tissue for 1 min (power density, 1562.5 W/cm^2^; fluence, 7 J/cm^2^) and the application was repeated five times (5 min for session). In addition, ZnC mouthwash was administered as a 10 mL mouthwash, three times daily after meals. Patients were instructed to keep the solution in the mouth for 3 min and to avoid eating or drinking for at least 2 h after each application, for a total duration of 1 month. The clinical course of the three cases is summarized in Table 2.

**Table 2:** Timeline summarizing treatment and clinical outcomes of three cases.

Timepoint	Case 1	Case 2	Case 3
Baseline	WHO grade III; VAS 8.7/NRS 8; lesion area 934 mm^2^	WHO grade III; VAS 0.2/NRS 3; lesion area 695 mm^2^	WHO grade II; VAS 7.1/NRS 7; lesion area 1088 mm^2^
T0	Start PBM + ZnC	Start PBM + ZnC	Start PBM + ZnC
T2	Marked pain reduction (VAS 1.7)	Stable low pain	Marked pain reduction (VAS 1.3)
T7	Stable improvement	57.6% lesion reduction (295 mm^2^)	100% lesion reduction
T14	89.9% lesion reduction (94 mm^2^); WHO grade I	Stable improvement	—
T21	Complete healing (100%)	Complete healing (100%)	—

### 2.4. Results

Two patients revealed a grade III OM and one patient a grade II OM, according to WHO classification. In days following treatment, a significant shift towards less severe stages was observed. No adverse events were observed during the follow‐up period.

#### 2.4.1. Case 1

Alomst 2 weeks after the start of treatment, consisting of two PBM sessions and the use of zinc‐carnosine mouthwash (10 mL, three times a day), a marked improvement in the clinical findings and symptoms was detected.

At 2 weeks of follow‐up, intraoral examination showed a significant reduction in lesion areas, with complete resolution of erosive/ulcerative lesions with only a thin residual erythematous band (Figure 2b). The patient also reported recovery of her ability to eat solid foods.

The symptoms reported and clinical signs observed allowed the mucositis to be classified as grade I according to the WHO classification.

#### 2.4.2. Case 2

Alomst 7 days after treatment, consisting of a two‐laser session and the use of use of ZnC mouthwash (10 mL, three times a day), the patient showed a favorable clinical response.

**Figure 5:**
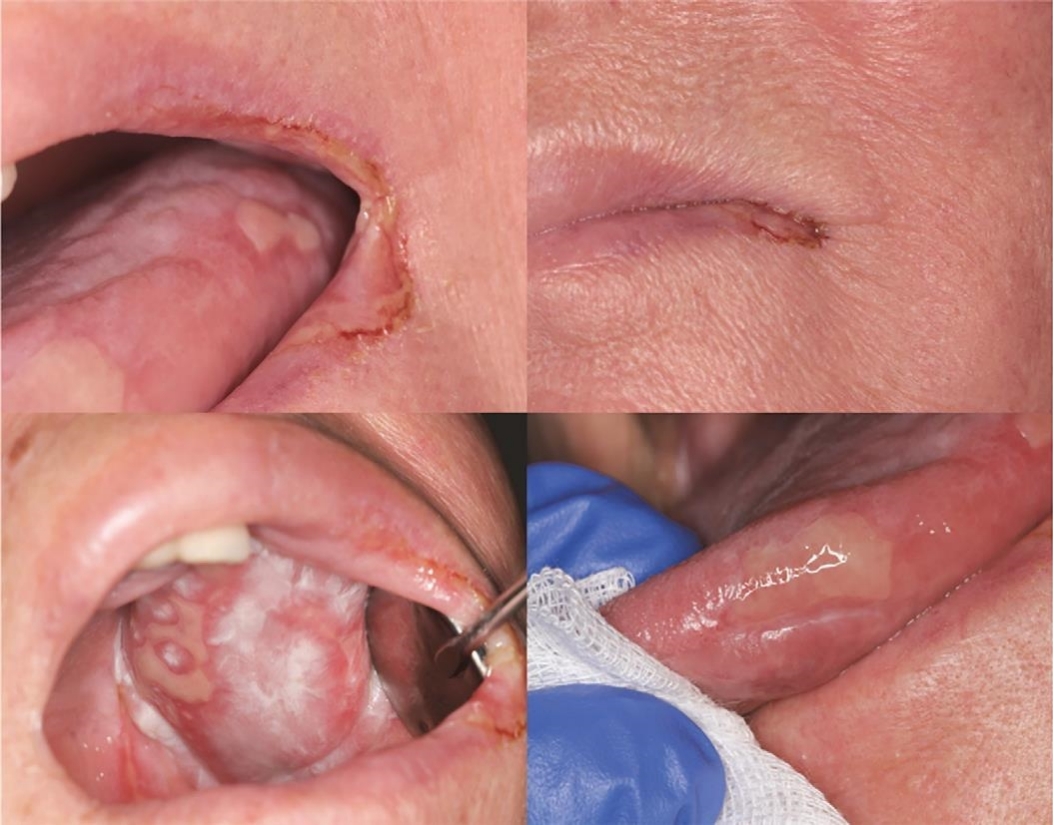
Case 2: follow‐up T7: reduction in the extent of the erosive/ulcerative areas following 1 week of combined therapy.

As illustrated in Figure 5, the lesions had begun a healing process, with a visible reduction in the extent of the erosive/ulcerative areas.

Alomst 3 weeks after treatment, consisting of four PBM sessions and the use of a ZnC mouthwash (10 mL, three times a day), the patient showed complete resolution of the OM with remission of painful symptoms.

**Figure 6:**
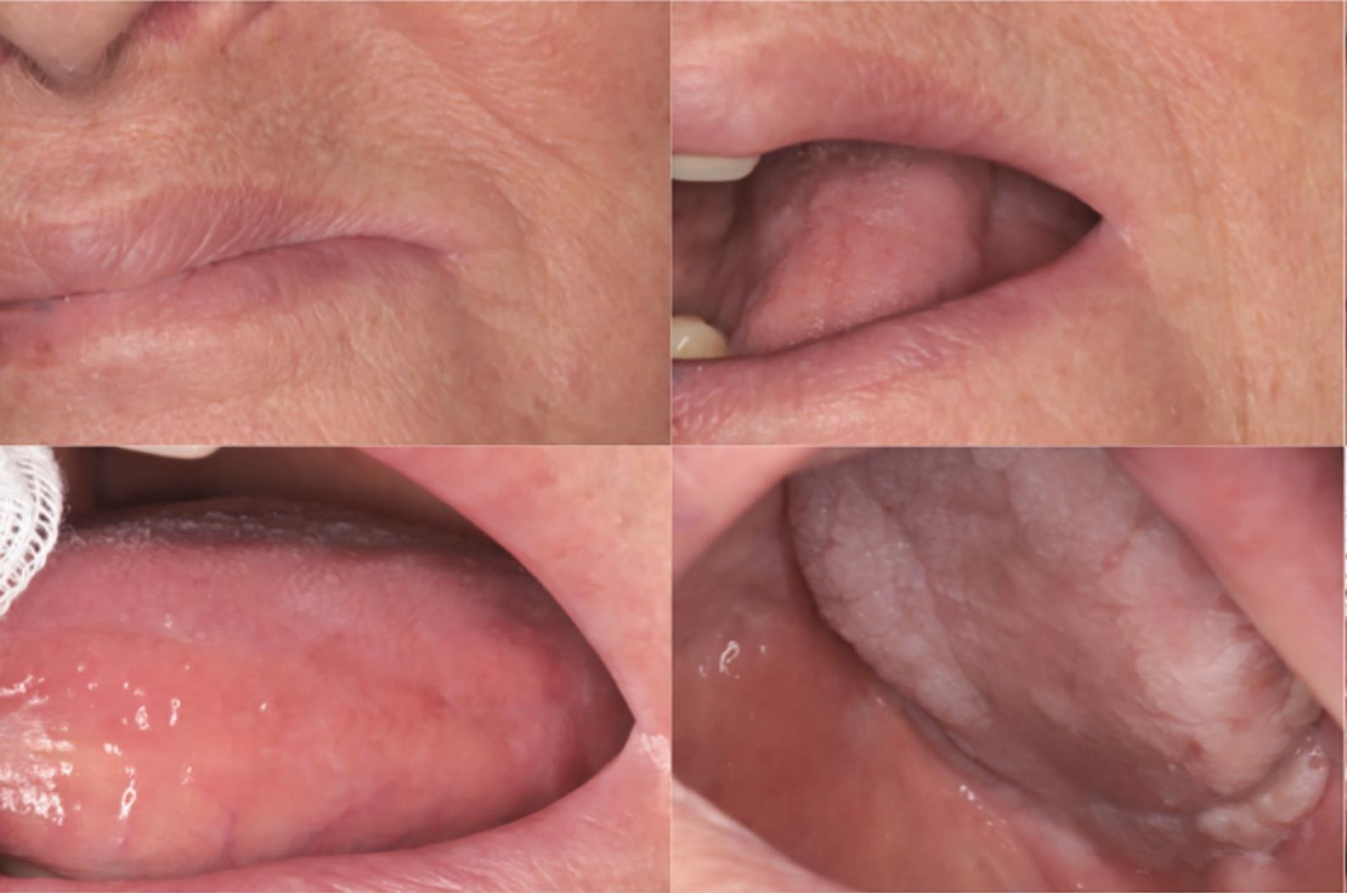
Case 2: follow‐up T21: complete healing: complete reepithelialization of previously ulcerated areas, with absence of visible mucosal defects (WHO grade 0).

As shown in Figure 6, complete healing of the RT‐related lesions was observed. The VAS and NRS values remained constant and of low intensity throughout the follow‐up.

#### 2.4.3. Case 3

One week after starting treatment, consisting of a single PBM session and ZnC mouthwash (10 mL, three times a day), complete healing of the erythematous lesions located on the right and left buccal mucosa was observed.

**Figure 7:**
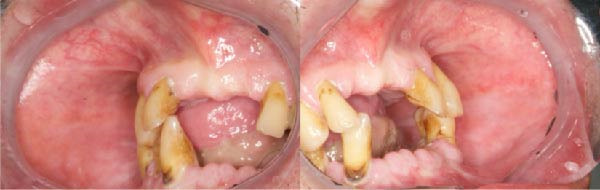
Case 3: follow‐up T7: complete clinical resolution of mucosal lesions and absence of inflammatory signs after combined ZnC and PBM therapy (WHO grade 0).

The patient reported no discomfort and no difficulty eating. As shown in Figure 7, at 7 days of follow‐up, intraoral clinical examination revealed total resolution of the OM.

### 2.5. Pain and Healing Assessment

Pain trajectories of the three cases of RIOM showed distinct patterns (Table 3). Case 1 and Case 3 presented with high baseline pain (VAS 8.7/NRS 8 and VAS 7.1/NRS 7, respectively) and showed a marked reduction, with the most significant decrease observed already at T2 (Case 1: VAS 1.7, 80.4% reduction; NRS 4, 50% reduction; Case 3: VAS 1.3, 81.7% reduction; NRS 4, 42.9% reduction), and minimal pain was observed at T14 (Case 1: VAS 0.2, 97.7% reduction; NRS 2, 75% reduction; Case 3: VAS 0.1, 98.6% reduction; NRS 1, 85.7% reduction). In contrast, Case 2 reported low pain throughout the treatment (T0 VAS 0.2/NRS 3), with values remaining largely stable up to T14 (VAS 0.3/NRS 2). Individual case data are illustrated in Figure 8a (VAS) and Figure 8b (NRS). In addition, all patients showed progressive improvement in oral feeding capacity, with restoration of tolerance to solid food during follow‐up. Furthermore, no concomitant antifungal agents, topical corticosteroids, or other specific treatments for OM were administered during the combined PBM and ZnC protocol.

Figure 8VAS scores (a) and NRS scores (b) over time in three cases.

**Figure:**
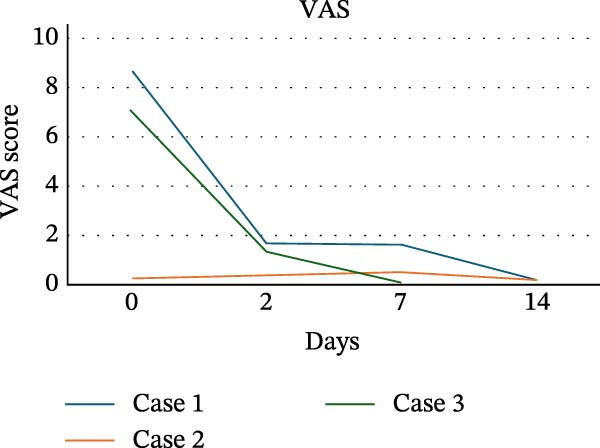
(a)

**Figure:**
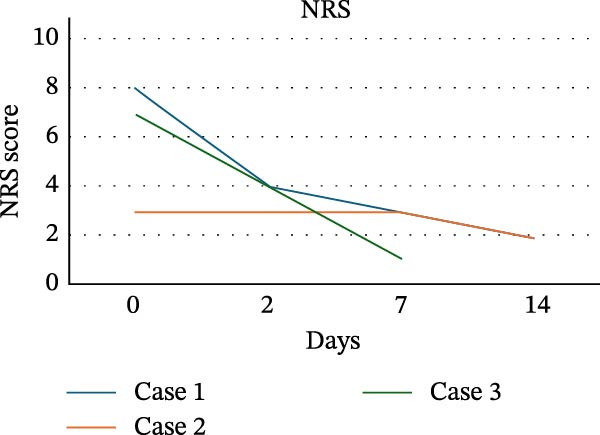
(b)

**Table 3:** VAS and NRS values.

Days	Case 1—VAS	Case 1—NRS	Case 2—VAS	Case 2—NRS	Case 3—VAS	Case 3—NRS
T0	8.7	8	0.2	3	7.1	7
T2	1.7	4	0.5	3	1.3	4
T7	1.6	3	0.5	3	0.1	1
T14	0.2	2	0.2	2	—	—

### 2.6. Evaluation of the Reduction in the Extension of Lesions Related to OM

The extension of the areas affected by OM and the percentage reduction were assessed by measuring the surface area using Fiji software before and after the start of treatment.

Measurements were taken at the sites most representative of the condition: in Case 1 at the palate, in Case 2 at the left lingual margin, and in Case 3 on the right buccal mucosa (Table 4).

**Table 4:** Areas values over time in follow‐up.

Case	T0	T7	T14	T21	Percentage of reduction
Case 1	934 mm^2^	—	94 mm^2^	0	89.9% at 14 days, 100% at 21 days
Case 2	695 mm^2^	295 mm^2^	—	0	57.6% at 7 days, 100% at 21 days
Case 3	1088 mm^2^	0	0	0	100% at 7 days

In Case 1, an area of 934 mm^2^ was recorded at time T0 and 94 mm^2^ at time T14, corresponding to a reduction of 89.9%. In Case 2, the measured area was 695 mm^2^ at T0, 295 mm^2^ at T7, with a reduction of 57.6% and complete resolution at T21. In Case 3, an area of 1088 mm^2^ was measured at the level of the right buccal mucosa, with a 100% reduction already at T7.

## 3. Discussion

The treatment of HNC depends on the stage at diagnosis and may include surgery, RT, targeted therapy, immunotherapy, and/or chemotherapy [19–21]. Despite planning aimed at protecting healthy tissue, radiation can damage nearby structures, causing OM, hyposalivation/xerostomia, dysphagia, and potentially very severe conditions such as osteoradionecrosis (ORN) [22–24].

RIOM was first identified and named in 1980 as a complication arising in cancer patients undergoing RT [25]. It manifests as a painful inflammation of the gastrointestinal mucosa, caused by the local damaging action of radiation, which can significantly reduce the patient’s quality of life and may lead to discontinuation of cancer treatment [26]. RIOM presents with a wide spectrum of clinical manifestations, varying greatly in severity. They range from the mildest forms, characterized by simple erythema of the mucosa, to the most severe pictures, in which extensive damage to the mucous membranes is observed, with atrophy, ulceration, and bleeding [27]. In addition to severe pain, patients may experience a range of additional symptoms that further aggravate the clinical picture: dysgeusia, xerostomia, and odynophagia, to more severe conditions such as malnutrition and dehydration, resulting in weight loss and risk of septic complications due to the impairment of epithelial and basement membrane barriers that normally protect tissues [28]. The severity of mucositis is influenced by the radiation type, total dose, and duration of treatment. In particular, even relatively low cumulative doses of 1000–2000 cGy, delivered at 200 cGy per day, can trigger the onset of RIOM [29].

Several clinical criteria have been reported for assessing RIOM, including those by WHO, Radiation Therapy Oncology Group/European Organization for Research and Treatment of Cancer (RTOG/EORTC Scale), and OM Assessment Scale (OMAS) [30]. The most used classification is that proposed by the WHO, which is based on an increasing numerical scale according to severity (Table 1). This scale evaluates the different degrees of clinical symptomatology, considering both objective signs and functional impact on the patient [31].

RIOM is a very common condition; in fact, about 40% of patients treated with chemotherapy develop this condition, and its incidence increases significantly in patients undergoing hematopoietic stem cell transplantation (HSCT), reaching 60%–85%. The data are even more alarming in patients with cancers of the head and neck region treated with RT combined with chemotherapy: in this group, mucositis affects up to 90% of cases. Among these patients, about 11%–19% will experience hospitalization and be forced to temporarily delay or suspend antineoplastic treatment [4, 32].

The Multinational Association of Supportive Care in Cancer (MASCC) and the International Society of Oral Oncology (ISOO) recommend various approaches for managing RIOM, including oral care, NSAIDs, mucosal protectants, growth factors, and analgesics [33]. Palifermin (KGF‐1) is the only FDA/EMA‐approved drug for preventing OM in patients undergoing high‐dose chemotherapy combined with total body irradiation (TBI) prior to HSCT. It promotes epithelial repair and has antioxidant, antiapoptotic, and anti‐inflammatory effects. Its use in HNC is limited due to high cost and potential tumor‐stimulating effects, highlighting the need for safer and more accessible alternatives [34].

The crucial role of managing cancer patients through dental treatment prior to head and neck RT and/or chemotherapy should not be underestimated. This reduces the risk of ORN, radiation caries, xerostomia, and OM. Preventive management of oral diseases reduces infections, ORN, decreases the risk of interrupting cancer treatment, and improves quality of life, highlighting the importance of preventive dental evaluation [35, 36].

The pharmacological properties of ZnC derive from the complementary actions of its two components. Zinc is an essential trace element required for cellular proliferation, epithelial regeneration, and wound healing. Deficiency in zinc is associated with delayed tissue repair, dermatological manifestations, and taste disturbances. L‐carnosine, on the other hand, is a naturally occurring dipeptide found in muscle tissue, with potent antioxidant and cytoprotective activities that support immune defense and have been implicated in the prevention of various metabolic and degenerative conditions.

By combining these two molecules in a chelated form, ZnC ensures improved stability, enhanced absorption, and sustained zinc release at the target site. This unique formulation provides therapeutic benefits that exceed those achievable with either zinc or carnosine administered alone, making ZnC a promising adjunct in the management of mucosal damage, particularly in the context of OM induced by RT.

Evidence from in vitro studies and animal models consistently highlights the protective role of ZnC on mucosal surfaces, suggesting its therapeutic potential in conditions characterized by chronic mucosal injury and oral surgical wound healing [37, 38]. These findings have prompted clinicians to explore the use of ZnC in the management of RIOM, where maintaining mucosal integrity and controlling pain represent major clinical challenges. In fact, several studies in the literature have investigated its efficacy in this specific setting, reporting encouraging outcomes in terms of both symptom control and mucosal healing, but also in the prevention of OM [2, 32, 39]. An additional advantage of ZnC lies in its compatibility with concomitant topical therapies, which facilitates its integration into personalized treatment regimens [6].

Protocols for PBM administration vary widely, with wavelengths ranging from 632.8 to 955 nm and energy delivery from 1.5 to 8.0 J/cm^2^, reflecting the need for protocol optimization depending on clinical context [40]. Clinically, it has proven useful in the management of various oral disorders by facilitating tissue repair and reducing pain. Several studies suggest that the diverse therapeutic effects of PBM make it a valuable adjunctive therapy for both the prevention and treatment of RIOM [41–43]. In addition, a recent review conducted by the mucositis study group of the MASCC/ISOO supports PBM for the prevention of OM and related pain in patients undergoing stem cell transplantation or head and neck RT, while evidence remains insufficient to provide guidelines for treating established OM or chemotherapy‐induced OM [44].

These preliminary results showed that the use of this new combined approach seems to highlight a positive impact on the management of patients with RIOM. The results obtained are supported by specific evidence: laser PBM promotes healing of oral wounds, increasing vascularization and reducing the production of pro‐inflammatory cytokines. Also, its analgesic effect is now well known in the literature. On the other hand, according to studies in the literature, the use of ZnC mouthwash has an important anti‐inflammatory and antioxidant effect, which reduces oral lesions by accelerating tissue healing processes. Our preliminary results are consistent with studies in the literature reporting beneficial effects of PBM and ZnC in reducing the incidence and severity of RIOM [12, 13, 45–48]. However, published studies analyze these two techniques separately. This study suggests that the use of a ZnC mouthwash appears to enhance the beneficial effects of laser PBM, contributing to greater reduction in painful symptoms and faster healing. Therefore, this combined approach could be a safe and feasible additional treatment for the management of RIOM. This case series provides a quantitative assessment of the progressive reduction in mucosal lesions using digital image analysis software and standardization of pain symptom recording using the VAS and NRS scales. However, there are some important limitations. The small number of patients enrolled and the lack of a control group reduce the strength of the evidence reported in this study. Furthermore, a combined approach does not allow for differentiation between the individual effects of PBM and ZnC. Therefore, these results should be interpreted with caution. Future research should focus on confirming these preliminary results through the design of a randomized controlled clinical trial with long‐term follow‐up to evaluate both the preventive and therapeutic potential of this combined therapeutic approach in patients undergoing head and neck RT.

## 4. Patient Perspective

Patients reported a marked and consistent reduction in pain symptoms after starting the combined approach and throughout the duration of therapy. The proposed protocol was well tolerated, with no adverse reactions reported and an adequate response to therapy.

## Author Contributions

**Pierpaolo De Francesco, Ilaria Giovannacci, Claudia Grondelli, Paolo Vescovi:** conceptualization, formal analysis, writing – original draft. **Pierpaolo De Francesco and Ilaria Giovannacci:** investigation, methodology. All authors: writing – review and editing.

## Funding

This research received no external funding. Open access publishing facilitated by Universita degli Studi di Parma, as part of the Wiley ‐ CRUI‐CARE agreement.

## Disclosure

All authors have read and agreed to the published version of the manuscript.

## Ethics Statement

This study was approved by the Area Vasta Emilia Nord Ethics Committee (Parma, Italy) (Protocol Number 40780; Code Number 500/2025/PARERE/AOUPR; September 29, 2025). The study was conducted in accordance with the Declaration of Helsinki.

## Consent

Informed consent was obtained from all subjects involved in the study.

## Conflicts of Interest

The authors declare no conflicts of interest.

## Supporting Information

Additional supporting information can be found online in the Supporting Information section.

## Supporting information


**Supporting Information** The study follows CARE guidelines, and the corresponding checklist is included as Supporting material.

## Data Availability

Data are available upon request from the authors.
