# Supplementary material for: A Case Series on the Combined Use of Zinc L‐Carnosine and Nd:YAG Photobiomodulation for Radiation‐Induced Oral Mucositis: Pain Reduction and Lesion Improvement
Source: Case Rep Dent. 2026 Apr 3;2026:7025093. doi: 10.1155/crid/7025093 (PMC13051797; doi:10.1155/crid/7025093)
Supplement: Supplementary file 1 — Supporting Information The study follows CARE guidelines, and the corresponding checklist is included as Supporting material. [file CRID-2026-7025093-s001.pdf]

CARE case report guidelines

CARE Checklist of information to include when writing a case report

(cc) BY-NC-ND

Topic	Item	Checklist item description	Reported on Line
Title	1	The diagnosis or intervention of primary focus followed by the words "case report"	1-2
Key Words	2	2 to 5 key words that identify diagnoses or interventions in this case report, including "case report"	17-18
Abstract (no references)	3a	Introduction: What is unique about this case and what does it add to the scientific literature?	22-27
	3b	Main symptoms and/or important clinical findings	27-29
	3c	The main diagnoses, therapeutic interventions, and outcomes	31-33
	3d	Conclusion—What is the main "take-away" lesson(s) from this case?	33-35
Introduction	4	One or two paragraphs summarizing why this case is unique (**may include references**)	52-56
Patient Information	5a	De-identified patient specific information.	163/175-176/206-207
	5b	Primary concerns and symptoms of the patient.	167-169/181-184/210-213
	5c	Medical, family, and psycho-social history including relevant genetic information	162-165/174-178/205-208
	5d	Relevant past interventions with outcomes	169-170/184-185/212-213
Clinical Findings	6	Describe significant physical examination (PE) and important clinical findings.	165-169/179-182/209-212
Timeline	7	Historical and current information from this episode of care organized as a timeline	168-170/182-185/211-213
Diagnostic Assessment	8a	Diagnostic testing (such as PE, laboratory testing, imaging, surveys).	165-167/179-181/209-210
	8b	Diagnostic challenges (such as access to testing, financial, or cultural)	
	8c	Diagnosis (including other diagnoses considered)	163-176-206
	8d	Prognosis (such as staging in oncology) where applicable	
Therapeutic Intervention	9a	Types of therapeutic intervention (such as pharmacologic, surgical, preventive, self-care)	230-233
	9b	Administration of therapeutic intervention (such as dosage, strength, duration)	234-241
	9c	Changes in therapeutic intervention (with rationale)	
Follow-up and Outcomes	10a	Clinician and patient-assessed outcomes (if available)	250-292
	10b	Important follow-up diagnostic and other test results	360-387
	10c	Intervention adherence and tolerability (How was this assessed?)	297-332
	10d	Adverse and unanticipated events	247-248
Discussion	11a	A scientific discussion of the strengths AND limitations associated with this case report	431-438
	11b	Discussion of the relevant medical literature **with references**.	422-431
	11c	The scientific rationale for any conclusions (including assessment of possible causes)	388-420
	11d	The primary "take-away" lessons of this case report (without references) in a one paragraph conclusion	431-434
Patient Perspective	12	The patient should share their perspective in one to two paragraphs on the treatment(s) they received	445-448
Informed Consent	13	Did the patient give informed consent? Please provide if requested 470-471	Yes [x] No [ ]
